# Self-measure of heart rate variability (HRV) and arrhythmia to monitor and to manage atrial arrhythmias: personal experience with high intensity interval exercise (HIIE) for the conversion to sinus rhythm

**DOI:** 10.3389/fphys.2014.00251

**Published:** 2014-07-08

**Authors:** David W. Young

**Affiliations:** HEAL ResearchChehalis, WA, USA

**Keywords:** heart-rate-variability, cardiac-arrhythmia-conversion, arrhythmia-reduction, premature-contractors, high-intensity-interval-exercise, HIIE, HRV-with-aging

## Introduction and background: benefits of self-measure

Self-Measure (SM) of heart rate variability (HRV) may provide a novel tool to monitor and to manage atrial arrhythmias. In the present article, I report my experience monitoring HRV and arrhythmias simultaneously, and how I discovered that acute episodes of high intensity interval exercise (HIIE) terminated atrial arrhythmias. I propose that SM of HRV may help identify therapies that retard the progression of arrhythmia toward atrial fibrillation (AFIB) and stroke with aging, starting at ages 45–55 years of age (Feinberg et al., 1995).

## Medical history

I am an 85 year old male. In 2009, I had the MAZE procedure during aortic valve replacement surgery to treat AFIB. The MAZE procedure was not effective, failing to prevent AFIB or to reduce the number of premature atrial contractions (PACs). Therefore, I recently underwent cryoablation of the left and right atrium to control AFIB and PACs, respectively. I am pleased to report that now, 3 months later, the cryoablation procedures have reduced the number of PACs and have eliminated AFIB. These procedures have given me a stable, predictable heart rhythm with the opportunity for an improved life. The residual premature atrial arrhythmias that occur in the evening have been managed successfully. I use 80 mg Sotalol during the day and 40 mg at night in combination with HIIE (Meyer et al., 2013) to convert atrial arrhythmias to a sinus rhythm when needed in the evening.

## Self-monitoring of my HRV

I began monitoring my HRV September 2008 using commonly available sensors. I measure the maximum sustainable resonant respiratory periodic HRV in bpm or RR Interval (RR-I in ms) for two or three nearly equal amplitude periods of HRV both at rest and during HIIE. Over the ensuing years, I found that arrhythmias disturbed my SM HRV as these arrhythmias increased non- monotonically to AFIB and stroke despite treatment with 80 mg of Sotolol, a beta-adrenergic receptor blocker administered twice a day. I personally confirm that increasingly frequent premature atrial complexes were associated with AFIB and adverse cardiovascular events (Chong et al., 2012).

SM led to a beneficial ablation of the right atrial for a dramatic reduction in the number of PACs, followed by left atrial ablation for AF. My initial cardiology consultation was less than favorable as this cardiologist would not look at my SM data and refused my request for ablation treatment. However, I was fortunate to find a second cardiologist who examined my HRV, HR, RR, ECG data, and agreed with my interpretation of these data, ultimately performing the ablations procedures that restored my cardiac rhythm.

I observed an abnormal HRV pattern after seizure. My HRV dropped from the typical 1.5–0.5 bpm, the wave form changed from sinusoidal to chaotic, and the HRV was not responsive to respiration. I also noticed that my HRV sometimes changes immediately after an ectopic pulse. These measures demonstrate the significant sensitivity and timeliness of the SM approach. These observations are coincident with the incidents and were detected at home rather than having to reproduce an irregularity on schedule at the doctor's office.

SM, therefore, is ideally suited to identify arrhythmias, (e.g., premature atrial, PAC; ventricular, PVC; or junctional, PJC, activations), and ECG anomalies, so that an appropriate medical facility and personnel can be selected to obtain optimal treatment. Early identification of patients at high risk of AFIB would enable more aggressive primary prevention and targeted intervention (Chong et al., 2012). In addition, SM of HRV could be used to evaluate the efficacy of a therapeutic intervention, thereby allowing the physician and patient to work together to find the optimal therapy, an example of “personalized medicine.” HRV SM could be used to determine the efficacy of exercise, resonant breathing, or medications to address non-lethal arrhythmias, enabling tailored treatment for each patient.

## SM for management of arrhythmia

Frequent PACs provide an example of a relatively benign arrhythmia that can become more serious with aging, since frequent premature atrial complexes predict new occurrences of atrial fibrillation and adverse cardiovascular events (Chong et al., 2012). One potential use of SM is PAC quantification in order to evaluate an individual's risk. Over the past decades, a vast amount of evidence has shown that almost any form of sustained or repetitive type of supraventricular arrhythmias including supraventricular ectopic beats can provoke myocardial dysfunction (Simantirakis et al., 2012). It has been known since 2006 that progressive aging of the heart is broadly associated with an increasing incidence of arrhythmias and disorders of the normal origin of the heartbeat (Jones, 2006). I, therefore, recommend that individuals in their late 50's or early 60's personally evaluate their ECG for abnormalities and their RR-I for obvious arrhythmias or use the CV Risk (next paragraph) as a guide which varies from a PAC period of 14.5 min to 6 s. Otherwise, clinically significant arrhythmias may be present for months or years, resulting in myocardial remodeling (Simantirakis et al., 2012).

## Premature atrial complexes and CV risk

Two studies found that a high premature pulse rate increases the risk for adverse cardiovascular events: Study (1) *One PAC or more every 14.5 min*, i.e., PACs >100 beats/day lowered the survival rate for 428 patients (without AF or structural heart disease) after 6.8 years 11%, when patients had frequent PACs (Chong et al., 2012). Study (2) Sixty Four participants in 14 years that initially had *10 PAC or more every minute* had 34 % combined CVD, CHD, HF, Stroke deaths while 7628 healthy participants without PACs had 7.8 % deaths for the same causes (Inohara et al., 2013).

## SM of HRV to detect my PACs

Recently, it was reported that a single session of HIIE improved the autonomic profile of CHF patients, leading to significant reductions of HR and arrhythmic events in a 24-h post training period (Guiraud et al., 2013). Thus, my experience with exercise is consistent with these findings, although I appear to have achieved satisfactory rhythm control more quickly than was reported. I have experienced many conversions that occurred during the first half of the HIIE period. However, there were two exercise sessions when a full, steady state conversion did not occur until 20 min after the exercise was complete. These two sessions were both conducted after the arrhythmia rate was high and later in the evening than usual. Nonetheless, the process is reliable, conversion having always been achieved, and almost always during the exercise period.

I typically experience two types of residual arrhythmia, one in the evening about 8:00 p.m. and one that initiates at 1 to 2:00 a.m. The rhythm patterns are different and recognizable. The 8:00 p.m. arrhythmias consists of a combination of single, double, and triple premature pulses along with sinus rhythm. The 2:00 a.m. rhythm rate varies the most: 88 bpm premature pulses mixed with a junctional escape rhythm bradycardia at 35 bpm and sinus rhythm @ 60 bpm. Incidentally, the 2:00 a.m. rhythm converts with the least exercise energy, e.g., running in place for a minute, but is also the most inconvenient since the conversion takes place in the middle of the night.

## Conversion of arrhythmia: evening-8 p.m., early morning-1:00 a.m.

**Figure 1:**
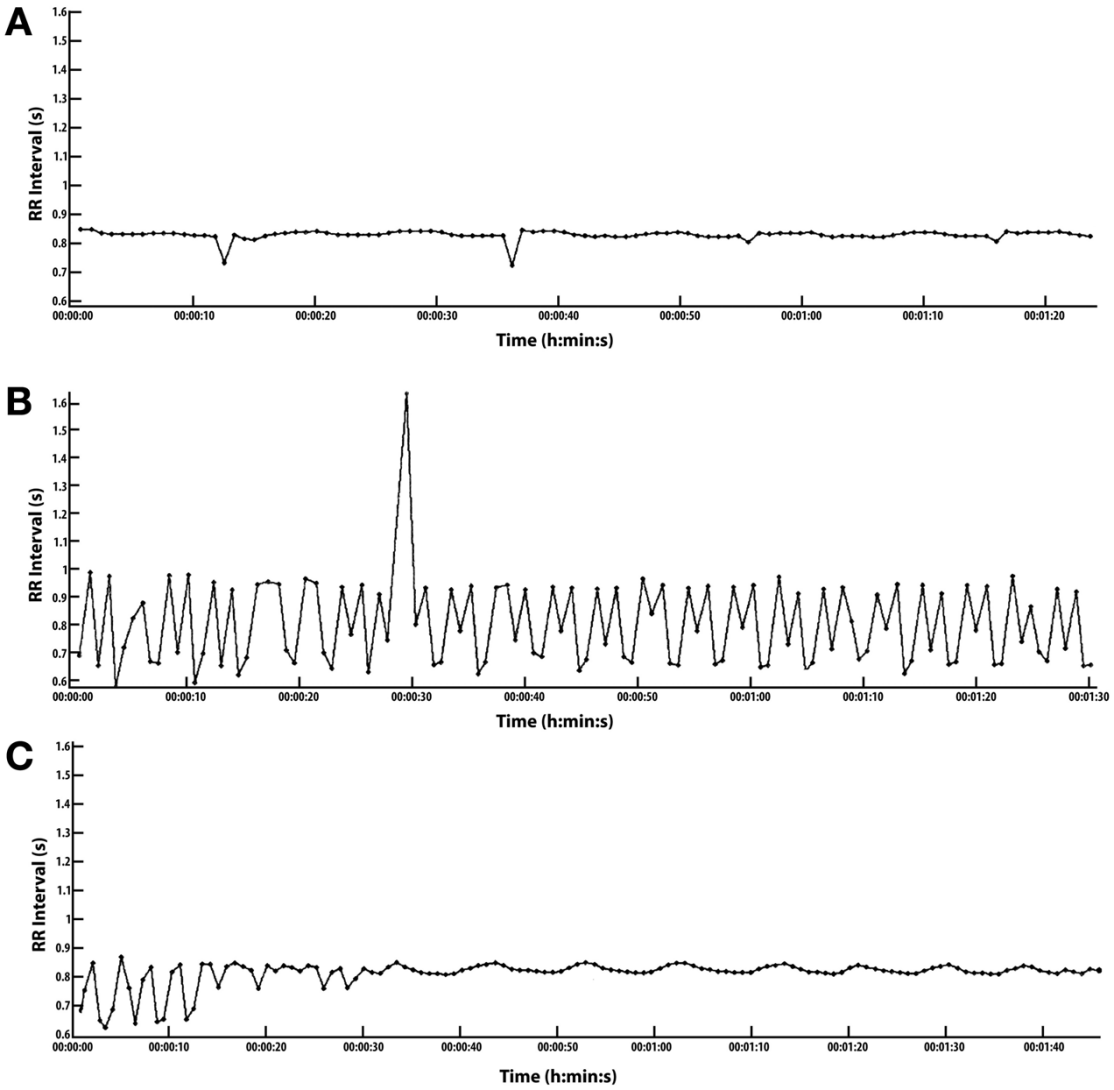
**Conversion of arrhythmia with high intensity interval exercise (HIIE). (A–C)** ordered from top of page to bottom. Scales are common: RR Interval (s) 0.6–1.6 s, Time (h:min:s) is 0 to 0:1:40 s. To convert RR-I in milliseconds to heart rate (HR), divide 60,000 by RR, i.e., 60,000/RR = HR, e.g., 60,000/600 = 100 bpm HR. **(A)** Conversion essentially complete. Recorded 5 min after exercise. HIIE starts after a 5 min warm up at 2.7 mph followed by 15 cycles of 1 min @ 3.7 mph and 1 min of rest for a total of 15 min of intense exercise @ 3.7 mph. **(B)** Arrhythmia recorded just prior to warm-up for HIIE. Arrhythmia composed of one PVC, a large number of multifocal premature contractions, and only 3 pulses joined along the typical sinus HRV at rest, and it takes about 13 pulses or 10 s to fully define one HRV wave or at least one half wave in 5 s. (HRV could not be measured in this case because there were too many PACs). Most of the arrhythmias fall within the following range: RR 0.65–1.0 ms, corresponding to a HR 60–92 bpm. **(C)** This is the cardiac recovery response during a 1 min rest period between HIIE intervals. This follows the 10th of 15 HIIE cycles after a 5 min warm up, extending the rest period from 1 min to 1.40 s for recording purposes. It takes 13 pulses typically to measure one HRV wave form in 10 s. With 13 pulses in 10 s, the SDNN can be calculated manually with an RR-I array of numbers. See Supplement. Results: 1st of 7 periods: HRV 2.8 bpm and Standard deviation of normal to normal beats (SDNN) 13 ms. Last of 7 periods: HRV 1.97 bpm and SDNN 10.9 ms.

The evening, 8:00 p.m., conversion of arrhythmia is the most dramatic and perhaps the most practical conversion as compared to the nighttime opportunities. The results of the evening conversion are shown in Figure 1. The evening conversion remains in sinus rhythm until about 2:00 a.m. the next morning when the lack of conversion seems to wake me without symptoms; that is, at least symptoms that I am aware of.

Even when dysrhythmic pulses occur in rapid sequence with few regular pulses, the conversion to sinus rhythm usually occurs before the HIIE is complete, i.e., only 15 min of cumulative intense exercise.

The arrhythmia occurs regularly between 1 and 3 a.m. On May 26th at 2:39 a.m. conversion was achieved in 1 min by trotting in place. Fortunately, sleep follows conversion quickly. The arrhythmia is probably present again sometime after conversion but these arrhythmias are always self-terminating in a little less than an hour after rising in the morning. Most incidents of arrhythmia occur at night, not during the day. I suspect that being horizontal a long time will also produce arrhythmia, but I have not confirmed this impression.

On one occasion on August 23rd, 2013, I was able to convert paroxysmal atrial tachycardia of 125 bpm. I applied intermittent experimental variations of the HIIE protocol and watched the regular 125 bpm rhythm first became irregular and then change from irregular to regular sinus in 1 h and 27 min. The total exercise period was probably less than 30 min. This SM result was encouraging.

Thus, as a result of using SM, I can confirm that Sotalol, HIIE, and paced breathing with a 10 s period manage my arrhythmia, and reduce my stress. By terminating these arrhythmias, I may also reduce irritation of the heart tissue, and perhaps extend the long-term outcome of the catheter ablation of AFIB (Ganesan et al., 2013).

## SM of ECG abnormalities

While using specific ECG abnormalities to suggest stroke risk may not be ready for prime time (Agarwal and Soliman, 2013), SM of the ECG could soon provide detailed insight for the individual. There are a number of published ECG abnormalities that are associated with an increased risk for stoke that an individual could use as part of their self-monitoring. Potential candidate markers for SM include: QRS/QT prolongation (Agarwal and Soliman, 2013), and frontal plane QRS-T angle (Aro and Huikuri, 2013).

## Sensors, leads, and displays: HRV, HR, RR, ECG

I often do SM with a 3 tier simultaneous, hybrid monitoring system that provides: (1) An immediate narrowband, real-time HRV waveform that is or is not consistent with respiration, defines normal or abnormal HR or HRV and is EMwave (www.heartmath.com) PC displayed. The EM wave is a narrow bandwidth sensor, being designed for HRV coherence in bpm so neither the amplitude nor timing of a PAC is correct. However, the evaluation is immediate. (2) The Wahoo HR monitor, chest or finger HR sensor displays real time RR-I numbers one at a time on the iPod HRV Tracker application. Thus, I can count how many regular pulses are displayed between premature pulses and thereby stratify risk and decide what action should be taken if there are too few normal pulses. The RR-I file is being stored during this time and then exported to Kubios (Tarvainen et al., 2013) for analysis. (3) The AliveCor.com ECG, is also being recorded and then evaluated for both premature and junctional rhythms. Recorded RR-I is evaluated with Kubios (Tarvainen et al., 2013) software for both arrhythmia and HRV bpm and SDNN-ms. Kubios RR graphics is used to find the PAC/PVC locations on the ECG.

When I have confidence that the ECG has identified the arrhythmia, I independently utilize the real time readout of the RR numbers on my iPod to count how many normal pulses I have between premature pulses, confirm that number on a Notepad table, and then fully analyze with Kubios. *This number is my most important SM variable I use on a day to day basis*; however, I always keep the ECG in mind.

Two of the three sensors are wireless and hand held and the PC is clip-wired to my ear, so it takes less than a 60 s to hook-up and start a recording.

## Conclusion

I have successfully used HRV SM to discover ECG abnormalities, to detect arrhythmias, and then to use this information to convert arrhythmias to an acceptable level of irregular heart rate with exercise and/or medication. SM can be used to monitor and to manage atrial arrhythmias and could thereby reduce a susceptible individual's AFIB and Stroke risk.

### Conflict of interest statement

The author declares that the research was conducted in the absence of any commercial or financial relationships that could be construed as a potential conflict of interest.

## Figures and Tables

**Table:** 

First of 7	830 to 865 ms,	HRV 2.8 bpm
RR-I periods:	72.3 to 69.5 bpm	SDNN 13
Last of 7	840 to 864 ms,	HRV 1.97 bpm,
RR-I periods:	71.4 to 69.48 bpm	SDNN 10.9
Resting-HR:		HRV 1.5 bpm,
		SDNN 7.7

## Supplementary material

SUPPLEMENT Figure 1 **(C)** HR and HRV exponential recovery from HIIE exercise: (Supplement, see expanded graphics). This details the recovery response beginning in about 32 s which coincides with the leading edge of the 1st of 7 waves. The Supplement presents this data graphically, concentrating the display on the HRV recovery waves one through seven. The HRV magnitude diminishes with time dramatically during this 1.0 min period. I have provided access to the digital RR-I files for Figure 1 as well as the ECG's for the same period in the Supplement so that the reader may conduct their own analysis.
