# Supplementary material for: Self-measure of heart rate variability (HRV) and arrhythmia to monitor and to manage atrial arrhythmias: personal experience with high intensity interval exercise (HIIE) for the conversion to sinus rhythm
Source: Front Physiol. 2014 Jul 8;5:251. doi: 10.3389/fphys.2014.00251 (PMC4085876; doi:10.3389/fphys.2014.00251)
Supplement: SUPPLEMENT Figure 1 — (C) HR and HRV exponential recovery from HIIE exercise: (Supplement, see expanded graphics). This details the recovery response beginning in about 32 s which coincides with the leading edge of the 1st of 7 waves. The Supplement presents this data graphically, concentrating the display on the HRV recovery waves one through seven. The HRV magnitude diminishes with time dramatically during this 1.0 min period. [file DataSheet1.DOCX]

**HRV Paper Supplementary Material.doc**

**SUPPLEMENTARY MATERIAL**

**Self-Measure of heart rate variability (HRV) to monitor and to manage atrial arrhythmias: Personal experience with high intensity interval exercise (HIIE) for the conversion to sinus rhythm**

D W Young

**SUPPLEMENTARY TEXT**

ALPHA, A THREE SENSOR SYSTEM OPERATION FOR HR HRV ECG RR

1. This is a snap shot of how the three sensors are used.
2. Turn on the sensors at about the same time.
3. The EMWave is a real time analog HRV wave form which gives me the opportunity to see my HRV-bpm amplitude build up in to a stable, resonant, maximized HRV, respiratory, near sinusoidal wave form, assuring the data being logged from the other sensors is appropriate and worthy of analysis.
4. The EMWave analog display tells me how well I am controlling the paced breathing and the air pressure in real time.
5. The EMWave has a moving slider to maintain the paced breathing. I set the pace for 10 seconds, 5 second inhale and 5 second exhale. Alternatively I use the second hand on my watch to maintain the pace.
6. However, if the EMWave shows an irregular, non-sinusoidal wave form, then I use the Alive ECG to identify the irregular pulse, the identity being after the fact. EMwave cannot be utilized for Self-Measure HRV while PACs or PVCs are present because a single pulse disturbs the trace for some 5 to 10 seconds due to the narrow bandwidth. The narrow band design for coherence is not compatible with the broad band needs of identifying a specific pulse, i.e. amplitude in Hz and time in milliseconds.
7. After I have recorded the ECG, I file it in pdf and then print it, studying the print out to be sure I didn’t miss anything. I look for premature complexes, what complexes they are and how often they occur. I am starting to look for prolonged QRS and QT duration. I measure HRV, HR modulation by counting mm between pulses using the RR-I markers at the bottom of the trace. This is easy for a HRV of 13 Hz for the middle aged, but not so easy for small HRV, e.g. 2. Hz for those in their 80’s. With an HRV variance of 65 to 67 bpm, i.e. 2 Hz, the differences in mm between the two pulses is only 0.7 mm.
8. The Wahoo HM on my chest or in contact with my fingers on a table radiates 2300 MHz to my IPod with an “ANT” a little less than a ¼ wave long, i.e. 0.7 inch. The display is part of a numeric HRV Tracker Application. The HR display is an average, but the RR-I is given in ms for each real time pulse. Connection status tells me if the ANT is connected and the serial number of the Wahoo HM that is active. After the recording I e-mail the RR-I file to myself with a Notepad file. From my PC, I recover the Notepad RR file, and send that file to Kubios for display and analysis.
9. All three sensors are sometimes used individually as well as collectively. However, I discard EMwave data if there are many PACs because the narrow bandwidth exaggerates the apparent number of PACs per second and under reports amplitude. I use the live HRV Tracker display in RR-I ms to detect arrhythmia real time and monitor progress while doing high intensity interval exercise. I memorize the conversion of RR numbers to HR in bpm because no one offers that convenience yet. The HR displayed on HRV Tracker is a slowly changing average not suitable for detecting PACs. I record both the intense activity and periods at rest for RR-I evaluation on Notebook direct or export to Kubios. I also use RR numbers to evaluate recovery time while using the tread mill.

HRV & AGING

This demographic view was provided by Peter Holzberger, MD 12-4-03. The Internist Approach to Atrial fibrillation, a view easily accessed by Google search.
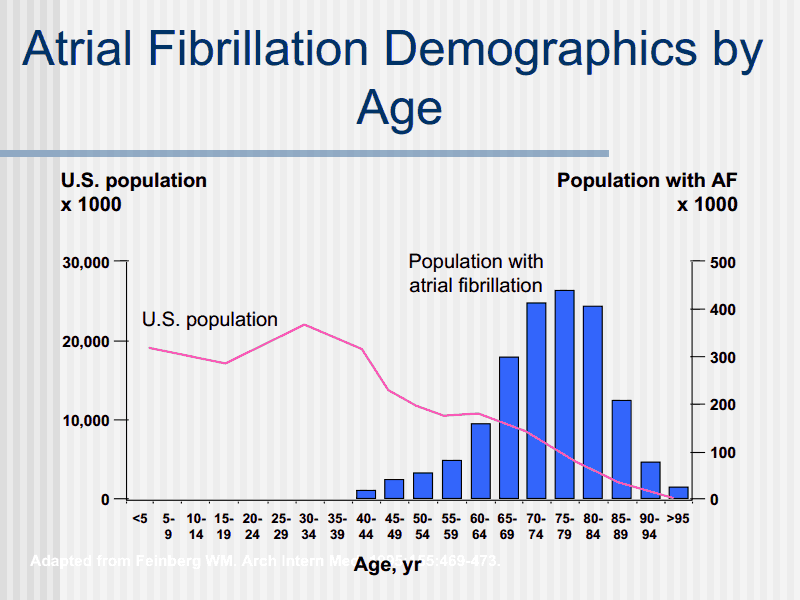


Estimated Numbers of People with Atrial Fibrillation in the US Population [1991 census data] (Feinberg et al., 1995)

Age, US Population [thousands] Estimated AF Population [thousands]

40-44 18,754 19

45-49 14,095 42

50-54 11,645 58

55-59 10,442 83

60-54 10,582 159

65-69 10,037 301

70-74 8,242 412

75-79 6,279 440

80-84 4,035 404

85-89 2,090 209

US Department of Commerce. Bureau of the Census. Statistical Abstract of the United States 1993. 113^th^ ed. Washington, DC: Bureau of the Census: 1993; 16 (data for 1991)

**SUPPLEMENT Fig.1 (C)**
