# Supplementary material for: Self-measure of heart rate variability (HRV) and arrhythmia to monitor and to manage atrial arrhythmias: personal experience with high intensity interval exercise (HIIE) for the conversion to sinus rhythm
Source: Front Physiol. 2014 Jul 8;5:251. doi: 10.3389/fphys.2014.00251 (PMC4085876; doi:10.3389/fphys.2014.00251)
Supplement: Supplementary file 3 [file DataSheet3.ZIP › ECG-20131231215508.pdf]

Patient: David Young, 4/26/28 (85yrs) **Comment:** 15tj

Recorded: Tuesday, December 31, 2013, 9:55:08 PM

Heart Rate: 72 bpm **Duration:** 1mins 12s

AliveCor™

Enhanced Filter, Mains filter: 60Hz Scale: 25mm/s, 20mm/mV

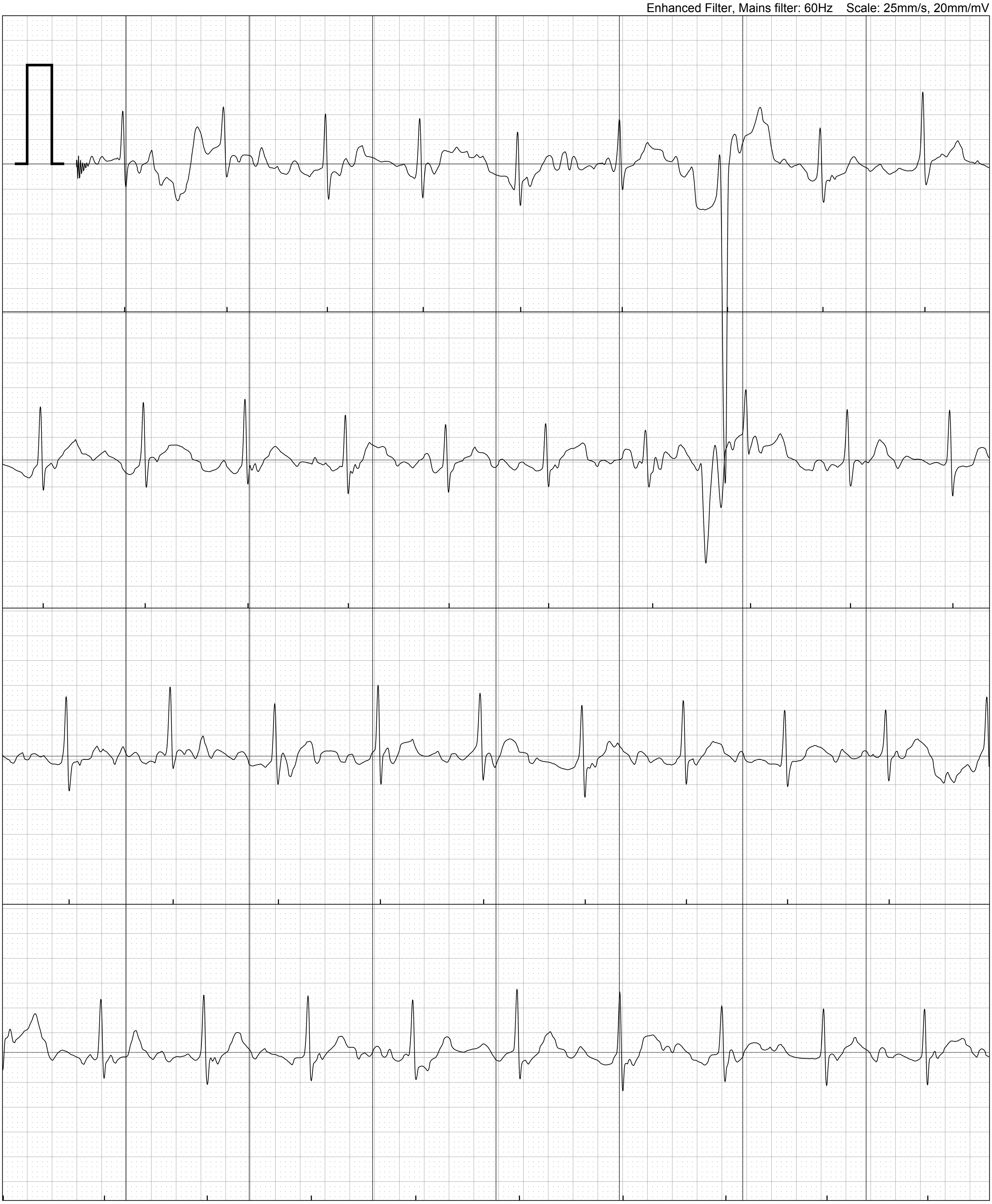

(c) Copyright 2012-2013, AliveCor Inc, AliveECG v1.5.2.1, Report v2.2.0, UUID: F802E25F-0BEA-4D0F-8D5A-FD58DA8DA86D

Patient: David Young, 4/26/28 (85yrs)
Comment: 15tj

Recorded: Tuesday, December 31, 2013, 9:55:08 PM

Heart Rate: 72 bpm **Duration:** 1mins 12s

AliveCor™

Enhanced Filter, Mains filter: 60Hz Scale: 25mm/s, 20mm/mV

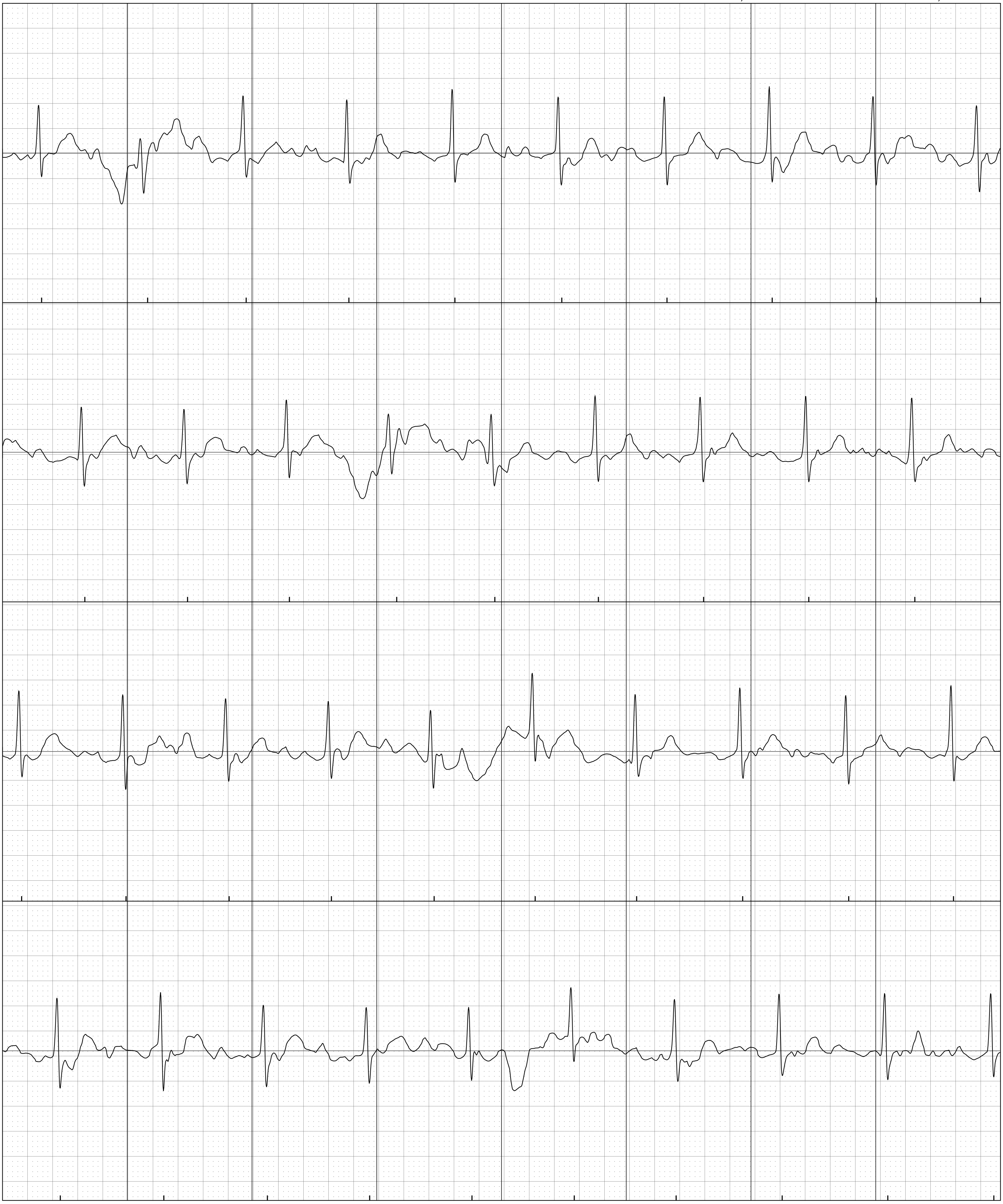

(c) Copyright 2012-2013, AliveCor Inc, AliveECG v1.5.2.1, Report v2.2.0, UUID: F802E25F-0BEA-4D0F-8D5A-FD58DA8DA86D

Patient: David Young, 4/26/28 (85yrs) **Comment:** 15tj
Recorded: Tuesday, December 31, 2013, 9:55:08 PM
Heart Rate: 72 bpm **Duration:** 1mins 12s

AliveCor™

Enhanced Filter, Mains filter: 60Hz Scale: 25mm/s, 20mm/mV

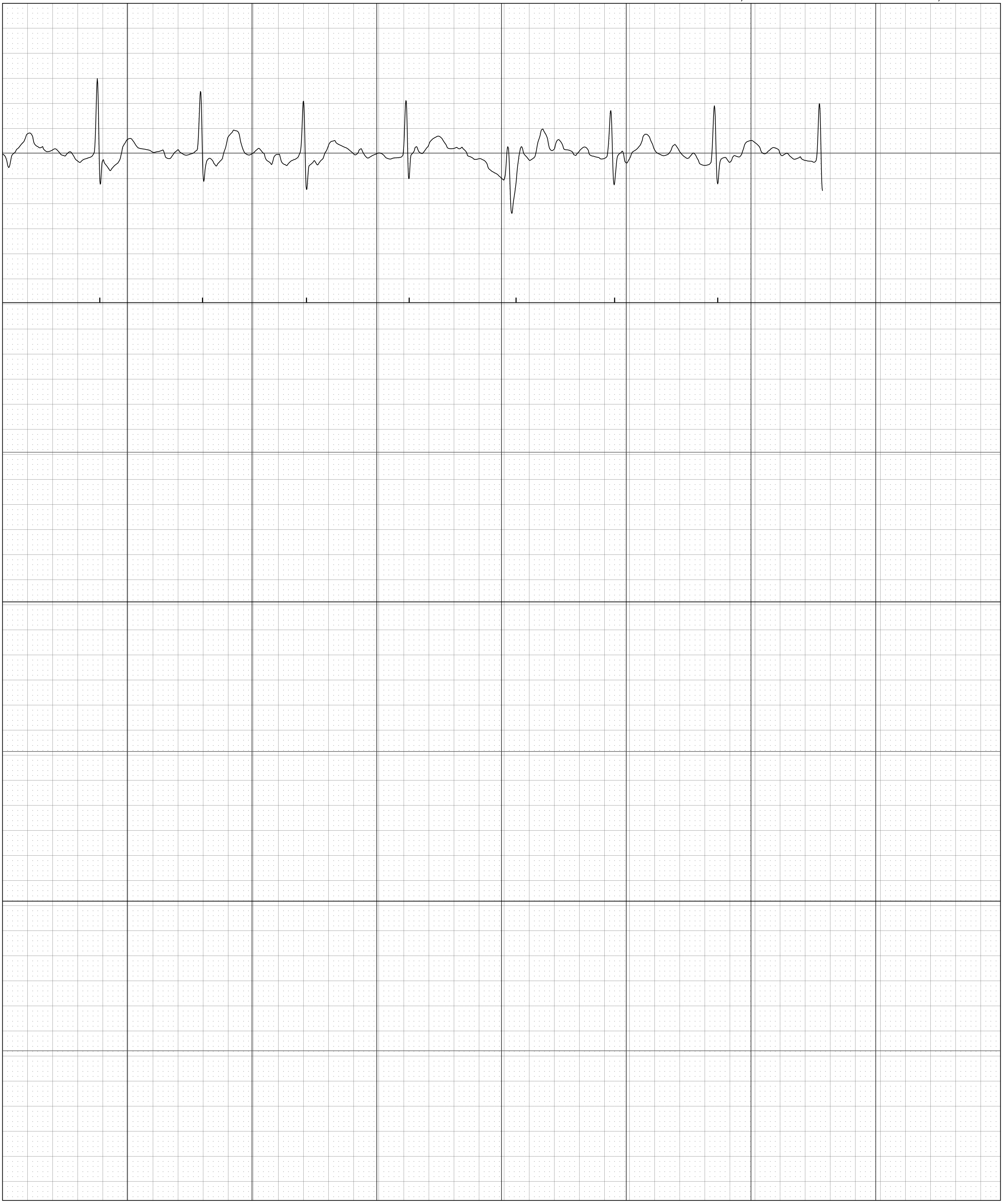

(c) Copyright 2012-2013, AliveCor Inc, AliveECG v1.5.2.1, Report v2.2.0, UUID: F802E25F-0BEA-4D0F-8D5A-FD58DA8DA86D
