# Supplementary material for: Self-measure of heart rate variability (HRV) and arrhythmia to monitor and to manage atrial arrhythmias: personal experience with high intensity interval exercise (HIIE) for the conversion to sinus rhythm
Source: Front Physiol. 2014 Jul 8;5:251. doi: 10.3389/fphys.2014.00251 (PMC4085876; doi:10.3389/fphys.2014.00251)
Supplement: Supplementary file 3 [file DataSheet3.ZIP › ECG-20131231221212.pdf]

Patient: David Young, 4/26/28 (85yrs)
Recorded: Tuesday, December 31, 2013, 10:12:12 PM
Heart Rate: 73 bpm **Duration:** 1mins 9s

Comment: 5 min after tread mill

Enhanced Filter, Mains filter: 60Hz Scale: 25mm/s, 20mm/mV

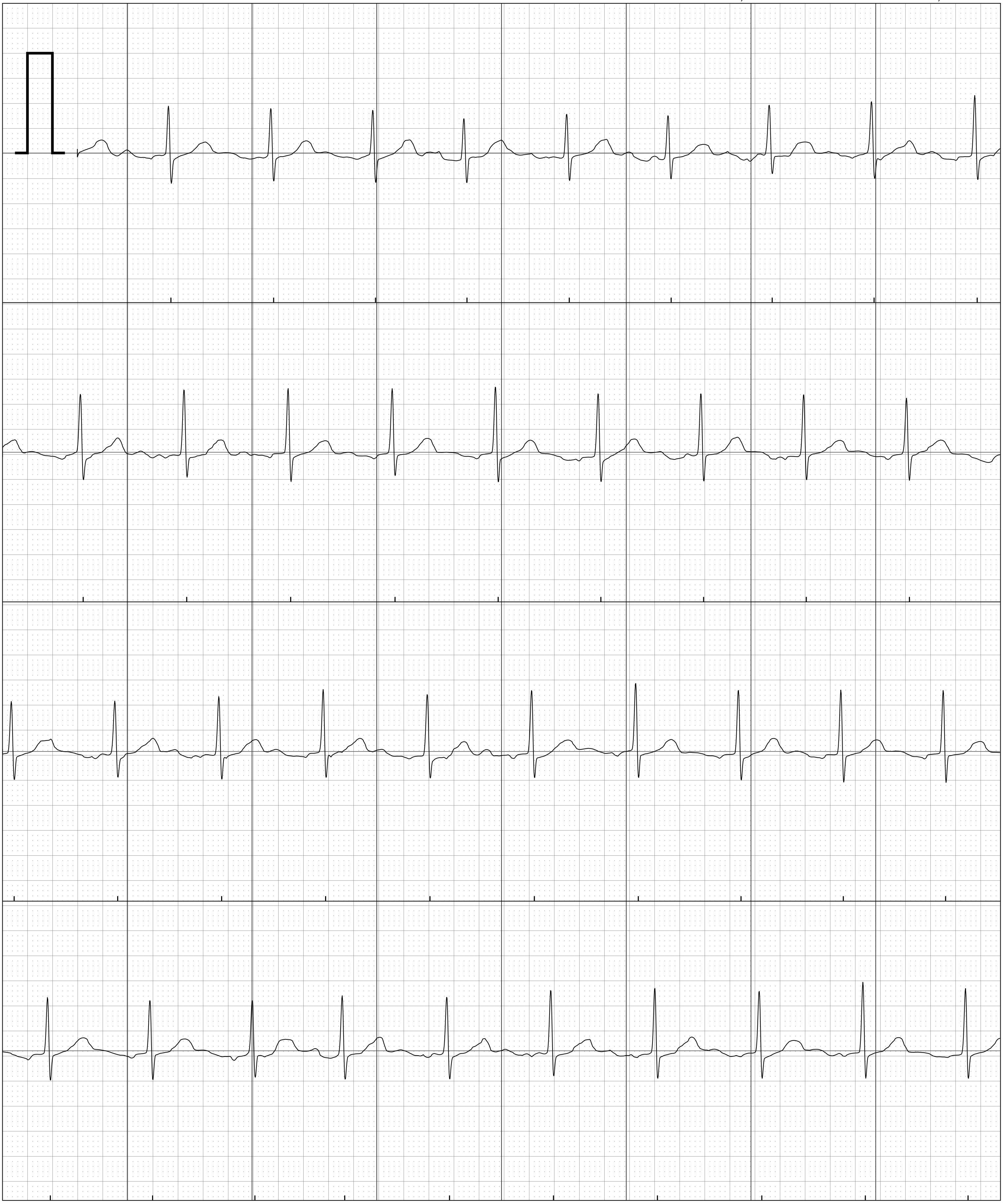

Patient: David Young, 4/26/28 (85yrs)
Recorded: Tuesday, December 31, 2013, 10:12:12 PM
Heart Rate: 73 bpm **Duration:** 1mins 9s
Comment: 5 min after tread mill

Enhanced Filter, Mains filter: 60Hz Scale: 25mm/s, 20mm/mV

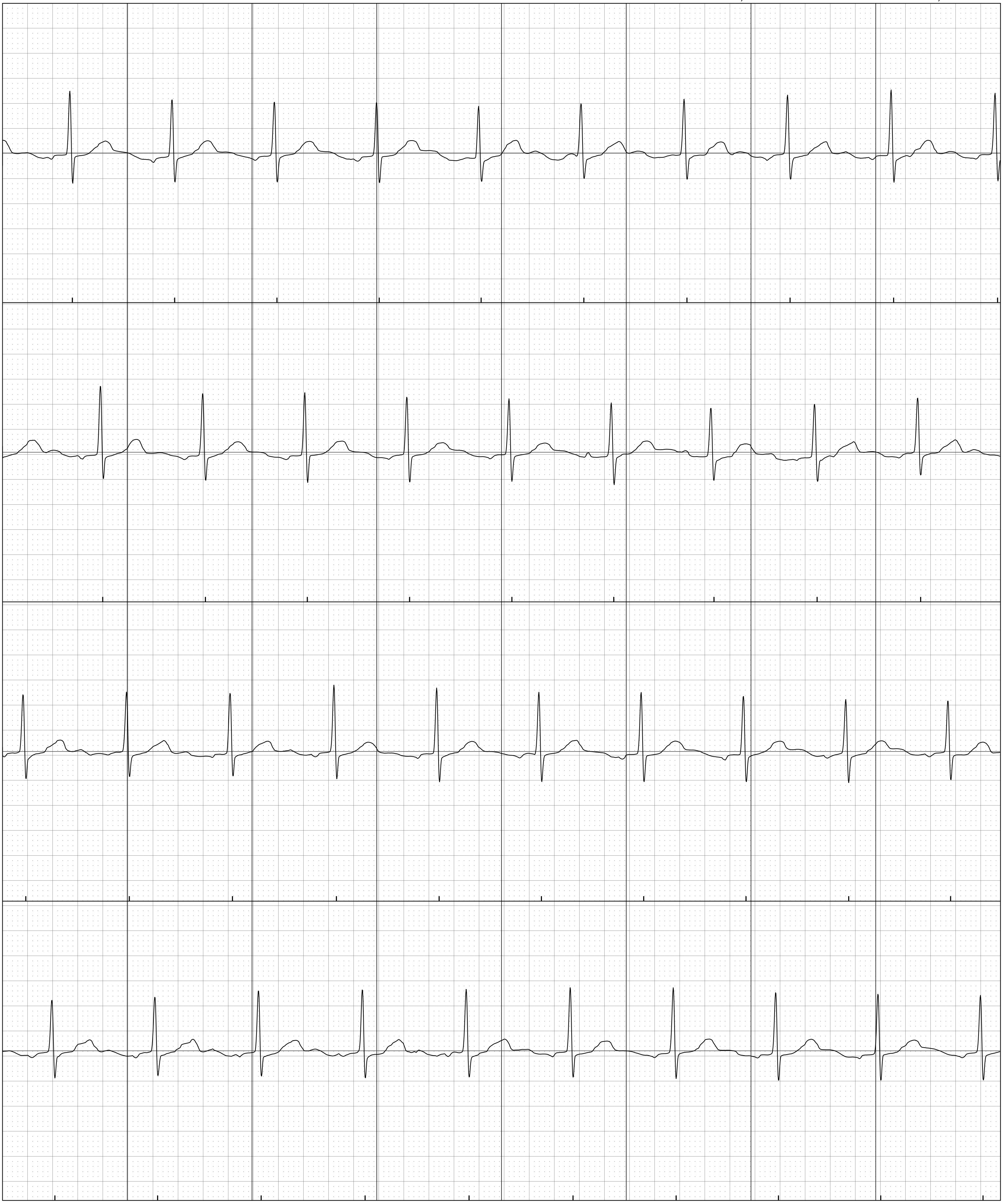

(c) Copyright 2012-2013, AliveCor Inc, AliveECG v1.5.2.1, Report v2.2.0, UUID: 1EF4E45F-A9FD-4490-8CDE-1E488CE72118

Patient: David Young, 4/26/28 (85yrs)
Recorded: Tuesday, December 31, 2013, 10:12:12 PM
Heart Rate: 73 bpm **Duration:** 1mins 9s

Comment: 5 min after tread mill

Enhanced Filter, Mains filter: 60Hz Scale: 25mm/s, 20mm/mV

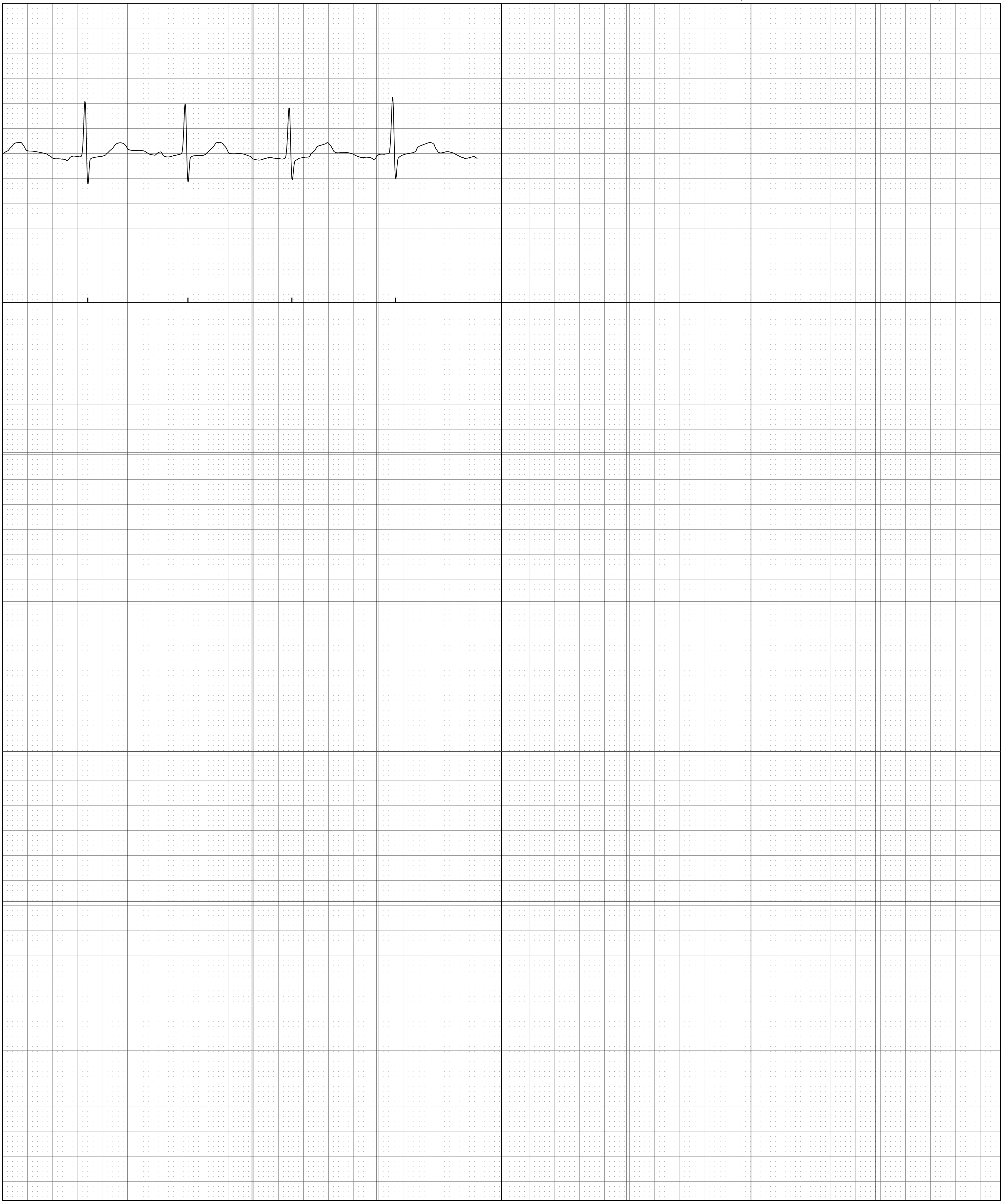

(c) Copyright 2012-2013, AliveCor Inc, AliveECG v1.5.2.1, Report v2.2.0, UUID: 1EF4E45F-A9FD-4490-8CDE-1E488CE72118
